# Editorial: New Frontiers in the Search of Antimicrobials Agents from Natural Products

**DOI:** 10.3389/fmicb.2017.00210

**Published:** 2017-02-21

**Authors:** Luis C. N. da Silva, Márcia V. da Silva, Maria T. dos Santos Correia

**Affiliations:** ^1^Programa de pós-graduação em Biologia Parasitária, Universidade CeumaSão Luís, Brazil; ^2^Departmento de Bioquímica, Centro de Biociências, Universidade Federal de PernambucoRecife, Brazil

**Keywords:** antibiotics, antivirulence, immunomodulation, action mechanism, bioactive compounds

Infectious diseases still one of the major causes of mortality and morbidity worldwide. The incidences of these infections are higher in communities exposed to inadequate sanitary conditions (commonly found in developing countries), and hospitalized and immunosuppressed individuals. This parasitic efficiency of microorganisms is due their high capacity to develop strategies to evade immune system and provoke host damage through virulence factor expression (Chan et al.; Hassan et al.; Al Atya et al.). In addition, these pathogens are able to acquire antimicrobial resistance by either drug-induced selection or drug-mediated mutagenesis (Castro et al.; Marini et al.; Morita et al.). Both cases result in diminution of antibiotic shelf time and make essential the search of new active compounds to be used in antimicrobial therapy. Natural products from micro-organisms, plants, animals, and algae have been proved to be excellent sources of antimicrobial compounds (Antoraz et al.; Deshmukh et al.; Santos et al.; Carter et al.; Lacerda et al.). The papers published in this Research Topic have proved this hypothesis as we illustrate bellow.

The production of antibiotics by microorganisms has been recognized for a long time. Several research groups have isolated new microbial species (bacteria, actinobacteria, fungi) able to produce new antimicrobial compounds or more productive strains (Antoraz et al.; Deshmukh et al.). In our Research Topic, active microorganisms were obtained from different niches since traditional sources—such as soil (Hassan et al.), plant (endophytes) (Santos et al.)—until unusual environments—such as Antarctic Ocean (Papa et al.), hypersaline habitats (Jose and Jebakumar), and mud wasp nests (Kumar et al.). Some papers also showed that active bacteria could be found associated to invertebrate organisms such as marine sponges (Graça et al.; Saurav et al.) and rhabditid entomopathogenic nematode (Deepa et al.). These microorganisms showed biotechnological aptitude as they produced compounds able to inhibit the growth of bacteria (Kumar et al.; de Oliveira et al.) and fungi (Kumar et al.; Kumar et al.).

Some bacteria were also able to inhibit virulence factors such as biofilm formation, toxins and proteases (Hassan et al.; Papa et al.; Saurav et al.). One example is nisin, a lantibiotic produced by *Lactococcus lactis*. Besides its activity against Gram-positive and Gram-negative planktonic cells of oral bacteria, nisin was able to impair multi-species biofilms (by inhibiting the biofilm formation or eradicating pre-formed biofilm). At anti-biofilm concentrations, nisin treatment did not result in toxicity toward human cells relevant to the oral cavity (Shin et al.). In another remarkable paper, the cis-2-decenoic acid (CDA) signaling network of *Pseudomonas aeruginosa* was revealed by microarray analysis. CDA is a signaling molecule involved in quorum-sensing. The authors also showed that this compound could disperse biofilms of *P. aeruginosa* alone or when combined with conventional antibiotics (Rahmani-Badi et al.).

An important effect against mycobacterial persister cells was shown for boromycin, a macrolide antibiotic produced by *Streptomyces antibioticus*. This compound targets mycobacterial transmembrane ion gradients and displays activity against both growing and non-growing drug tolerant cells. Its action was not related to resistance induction (Moreira et al.). Another elegant paper employed, for the first time, the phenylalanine/tyrosine ammonia lyase enzyme produced by *Rhodotorula glutinis* in the bioconversion of L-tyrosine methyl ester (L-TM) to the methyl ester of para-hydroxycinnamic acid (p-HCAM), an antibacterial product (MacDonald et al.). Finally, the improvement of antibiotic production through optimization of culture conditions was also demonstrated by a couple of papers (Kumar et al.; Rajeswari et al.).

Plants derived compounds are also attractive candidates for bioprospecting programs due their high chemical diversity which results in the inhibition of a range of microbial pathways (Santos et al.). Plants have been widely used by traditional communities due to their supposed medicinal properties. This popular knowledge has directed a lot of researches aiming to provide scientific evidence of these actions (Zhang et al.; Bezerra dos Santos et al.; Tiwari et al.; Saritha et al.; Silva et al., 2016). In other hand, some plants that are not used in folk medicine are also source of bioactive compounds. The papers published in this Research Topic discussed the action of different plant derived agents such as plant extracts (Saritha et al.; Silva et al., 2016), essential oils (Islamuddin et al.; Monte et al.; Hyldgaard et al.; Magi et al.), proteins (Silva and Correia; Patriota et al.), polyphenols (Stevens et al.; Taleb et al.), and triterpenoids (Wu et al.). The antimicrobial activity of these plant compounds was further characterized using alternative infections models such as *Caenorhabditis elegans* (Eng and Nathan) and Zebrafish embryo (Stevens et al.).

Another emergent topic in antimicrobial field is the research using honey as source of bioactive molecules. Indeed, honey has been used in traditional medicine to treat infections (Carter et al.). The multiple therapeutic properties of honey are due to its chemical diversity (sugars, peptides, proteins, hydroxymethylfurfural are examples of bioactive compounds) (Laallam et al.). One of these studies lead to the isolation of glycoproteins from honey which showed sequence identity with the Major Royal Jelly Protein 1 (MRJP1) which is composed by three antimicrobial peptides: Jelleins 1, 2, and 4. These glycoproteins exhibit a broad spectrum activity against multi-drug resistant clinical isolates (Brudzynski et al.).

Silva et al. discussed the use of antimicrobial peptides (AMP) from different sources (including microorganisms, insects, amphibians, plants, and humans) in the development of optical and dielectric sensors for microbial detection. Due the advances in nanotechnology, different nanostructured platforms containing AMP have been obtained resulting in biosensors with increased efficiency. These AMP-based biosensors could be employed for microbial diagnosis and for ensure the quality of different products such as water, food, and cosmetic.

Regarding the protozoal infections, the use of peptide from host insects as drug against *Leishmania* sp., *Plasmodium* sp., and *Trypanosomes* was reviewed by Lacerda et al. These authors also discussed the application of transcriptome analysis to prospect new peptides from non-host insects. Other paper demonstrated the leishmanicidal activity of *Piper nigrum*, a well-known medicinal plant. The bioactive fractions are able to induce apoptosis in promastigote forms of *Leishmania donovani*. Moreover, these fractions exhibited therapeutic action against experimental leishmaniasis which was related to their immunostimulatory potential (targeting Th1 pathway; Chouhan et al.). Anti-leishmania action was also evaluated to β-nitrostyrenes, a rare class of reddish brown compounds with antimicrobial activity which has been isolated from *Streptomyces lavendulaea* (2,4-dihydroxy-β-nitrostyrene) and Indian mangrove plant *Sonneratia acids* Linn. F (2-nitro-4-((E)-2-nitrovinyl)phenol). Some β-nitrostyrenes derivative compounds (synthesized using Henry reaction) were found to selectively inhibit promastigotes and amastigotes forms of *L. donovani* (Shafi et al.).

The antiviral properties of Cameroonian medicinal plants against hepatitis C virus (HCV) were also evaluated. The plants were selected based in an ethnobotanical survey which revealed that several plants from Western region of Cameroon have been used to treat liver-related disorders. Three plants (*Trichilia dregeana, Detarium microcarpum*, and *Phragmanthera capitata*) were found as source of anti-HCV compounds and the activity was related to inhibition of HCV entry without affect viral replication or secretion (Galani et al.). Other paper showed the potential use of dryocrassin ABBA against amantadine-resistant H5N1 avian influenza virus (HPAIV). Dryocrassin ABBA is a phloroglucinol derivative isolated from *Rhizoma Dryopteridis Crassirhizomatis*. This compound increased the survival rates of HPAIV virus infected mice, decreased lung index and virus loads. It also exhibited immunomodulatory action inhibiting pro-inflammatory cytokines (IL-6, TNF-α, and IFN-γ) and increasing anti-inflammatory cytokines (IL-10 and MCP-1).

Taken together, all these papers illustrate the versatility of natural products and highlight the importance of developing new prospection tools to impulse the discovery of new compounds. These molecules could be used as drug leads and also provide more insights for target discovery.

## Author contributions

All authors listed, have made substantial, direct and intellectual contribution to the work, and approved it for publication.

### Conflict of interest statement

The authors declare that the research was conducted in the absence of any commercial or financial relationships that could be construed as a potential conflict of interest.
